# Effectiveness of outpatient geriatric evaluation and management intervention on survival and nursing home admission: a systematic review and meta-analysis of randomized controlled trials

**DOI:** 10.1186/s12877-023-04036-4

**Published:** 2023-07-07

**Authors:** Pei-Chia Yen, Yu-Tai Lo, Chih-Cheng Lai, Ching-Chi Lee, Ching-Ju Fang, Chia-Ming Chang, Yi-Ching Yang

**Affiliations:** 1 Department of Family Medicine, Kuo General Hospital, No.22, Sec.2, Min Sheng Road, West Central Dist, Tainan, 700 Taiwan; 2 Department of Family Medicine, National Cheng Kung University Hospital, College of Medicine, National Cheng Kung University, No. 138, Sheng Li Road, North Dist, Tainan, 704 Taiwan; 3 Department of Geriatrics and Gerontology, National Cheng Kung University Hospital, College of Medicine, National Cheng Kung University, No. 138, Sheng Li Road, North Dist, Tainan, 704 Taiwan; 4 Department of Internal Medicine, Chi-Mei Medical Center, No.901, Zhong Hua Road, Yongkang Dist, Tainan, 710 Taiwan; 5 Clinical Medicine Research Centre, National Cheng Kung University Hospital, College of Medicine, National Cheng Kung University, No. 138, Sheng Li Road, North Dist, Tainan, 704 Taiwan; 6 Department of Secretariat, National Cheng Kung University Hospital, College of Medicine, National Cheng Kung University, No. 138, Sheng Li Road, North Dist, Tainan, 704 Taiwan; 7 Medical Library, National Cheng Kung University, No. 1, University Road, East Dist, Tainan, 701 Taiwan; 8 Department of Medicine & Institute of Gerontology, College of Medicine, National Cheng Kung University, No. 138, Sheng Li Road, North Dist, Tainan, 704 Taiwan; 9 Department of Family Medicine, College of Medicine, National Cheng Kung University, No. 138, Sheng Li Road, North Dist, Tainan, 704 Taiwan

**Keywords:** Outpatients, Geriatric assessments, Mortality, Nursing home, Meta-analysis

## Abstract

**Background:**

The benefit of inpatient comprehensive geriatric assessment on patient survival and function has been demonstrated among frail older patients. However, the influence of outpatient geriatric evaluation and management (GEM) on clinical outcomes remains debated. This study aimed to update the research evidence detailing the effect of outpatient GEM on survival and nursing-home admission through a comparison with conventional care.

**Methods:**

Cochrane Library, EMBASE, and MEDLINE databases were searched up to January 29th, 2022, to identify randomized controlled trials (RCTs) including older people over age 55 that compared outpatient GEM with conventional care on mortality (primary outcome) and nursing-home admission (secondary outcome) during a follow-up period of 12 to 36 months.

**Results:**

Nineteen reports from 11 studies that recruited 7,993 participants (mean age 70–83) were included. Overall, outpatient GEM significantly reduced mortality (risk ratio (RR) = 0.87, 95% confidence interval (CI) = 0.77–0.99, *I*^2^ = 12%). For the subgroup analysis categorized by different follow-up periods, its prognostic benefit was only disclosed for 24-month mortality (RR = 0.68, 95% CI = 0.51–0.91, *I*^2^ = 0%), but not for 12- or 15 to 18-month mortality. Furthermore, outpatient GEM had significantly trivial effects on nursing-home admission during the follow-up period of 12 or 24 months (RR = 0.91, 95% CI = 0.74–1.12, *I*^2^ = 0%).

**Conclusions:**

Outpatient GEM led by a geriatrician with a multidisciplinary team improved overall survival, specifically during the 24-month follow-up period. This trivial effect was demonstrated in rates of nursing-home admission. Future research on outpatient GEM involving a larger cohort is warranted to corroborate our findings.

**Supplementary Information:**

The online version contains supplementary material available at 10.1186/s12877-023-04036-4.

## Background

Modern medical science and evidence-based clinical guidelines focus on a single disease. Consequently, care for older adults tends to be duplicative, disjointed, and sometimes even harmful [1]. An increasing older population merits integrated and age-friendly healthcare services providing quality geriatric care. Comprehensive geriatric assessment (CGA) is a “multidimensional, multidisciplinary process that identifies medical, social, and functional needs, and the development of an integrated care plan to meet those needs” for individuals aged ≥ 55 years who require frequent acute medical care [2]. CGA is supported by considerable evidence to facilitate coordinated care for older patients with frailty and complex needs [3–5]. Different geriatric models based on CGA have been developed depending on various healthcare settings, including inpatient, in-home, and outpatient CGA [3].

Numerous systematic reviews and meta-analyses have demonstrated the effectiveness of CGA in various healthcare settings. Inpatient CGA improves patient survival and function at home and reduces the length of hospital stay among frail older patients [4, 6]. It also decreases the likelihood of nursing-home admission in such patients [7]. However, the effect of in-home CGA, namely outpatient geriatric evaluation and management (GEM) on reducing nursing-home admissions remains debated. Some investigations indicated that outpatient GEM effectively reduce mortality in the general older population [8, 9], but a meta-analysis consisting of nine randomized controlled trials (RCTs) conducted in the United States demonstrated a negligible benefit of outpatient GEM on survival [10].

Recently, several RCTs have expanded the reexamination of the influence of outpatient GEM on clinical outcomes. Fletcher et al. designed a large, population-based clustered RCT of outpatient GEM versus conventional care for individuals aged ≥ 75 years; they found that outpatient GEM had a trivial effect on mortality, hospitalization, or admission to other institutions [11]. Ekdahl et al. conducted an RCT comparing outpatient GEM and conventional care in older individuals (≥ 75 years) with three or more comorbidities and frequent inpatient admissions during the year before the study started [12]. They found that outpatient GEM resulted in longer survival and shorter hospitalization but no significantly higher costs. However, the evidence describing the beneficial effects of outpatient GEM programs in preventing nursing-home admission is limited.

Our aim in this study was to conduct a meta-analysis of research evidence detailing the effectiveness of outpatient GEM programs on survival, updated by Kuo et al.’s 2004 article, [10] and to explore nursing home admission among older individuals compared to conventional care.

## Methods

### Registration

The systematic review was reported in accordance with the 2020 PRISMA Statement [13] (Table S1), ensuring transparency and complete reporting. The research methods outlined in the Cochrane Handbook for Systematic Reviews [14] of Interventions were used, and the review was registered in PROSPERO (CRD 42,022,355,485).

### Search strategy and study selection

Three databases, namely Embase (Elsevier), MEDLINE (Ovid, including Epub ahead of print, in-process, and other non-indexed citations), and Cochrane Library (including clinical registers from World Health Organization International Clinical Trials Registry Platform and US ClinicalTrials.gov), were searched for relevant RCTs, with no language restrictions, from their inception dates to January 29th, 2022. The reference lists of the eligible articles were also reviewed to identify additional studies for possible inclusion.

Three key concepts, namely aged, outpatient, and GEM, were used in the search, including 39 synonyms in total and controlled vocabulary (10 Emtree terms and 8 MeSH terms). We applied highly sensitive search filters to identify RCTs. The complete search strategy is presented in Table S2.

### Eligibility criteria

RCTs that assessed the effects of outpatient GEM on survival and nursing-home admission were considered eligible. The included trials met the predefined PICO (population, interventions, comparison, and outcome) framework: (i) Population: older individuals (≥ 65-year-old) with or without frailty, individuals (≥ 55-year-old) with impaired activities of daily living (ADL), frailty, multiple comorbidities, and/or those with high healthcare services utilization in the previous year; (ii) Intervention: outpatient GEM led by geriatricians with a multidisciplinary team; (iii) Comparison: conventional care or standard care without involvement of geriatrician; and (iv) Outcome: number of patients with mortality (primary outcome) and nursing-home admission (secondary outcome) during the follow-up period of 12 to 36 months.

### Exclusion criteria

We excluded trials that fell into the following criteria: (i) unavailable outcomes; (ii) non-RCTs; (iii) incomplete in-person geriatric evaluation (i.e., carried out by telephone, simplified patient-filled questionnaire, screenings without full range assessments of geriatric syndromes, including medical, psychosocial, and functional capabilities) or the lack of follow-up management according to the initial assessment; (iv) GEM performed in settings other than outpatient clinics (i.e., assessments performed in inpatient departments, emergency departments, acute care units, home-care settings, or long-term care facilities); (v) GEM not led by geriatricians; and (vi) ongoing studies.

### Data extraction

After excluding duplicate studies in Endnote 20.2, two authors (P-C, Y and Y-T, L) independently assessed the study design, participants, interventions, and outcomes by screening the titles and abstracts before conducting independent full-text reviews. Disagreements were resolved by a between-screen discussion to reach a consensus. A third author (C-C, L) was consulted if consensus could not be reached. For the enrolled RCTs, the captured information included the author, publication year, patient characteristics (i.e., sex and age) and sources, study settings and locations, numbers of participants in the GEM and usual care groups, follow-up periods, numbers of patients with mortality and further nursing-home admission. The authors were contacted for further information if these variables were unavailable. Multiple reports with different periods of follow-up from the same study were identified manually and associated with each other by the review authors (P-C, Y and Y-T, L) and double-checked. Clinical registers, abstracts, or protocols and journal articles with identical study population were collated. Data were extracted from each report separately, then combine information in a data collection form.

### Assessment of risk of bias

Two researchers (P-C, Y and Y-T, L) independently rated the risk of bias using the revised Cochrane risk-of-bias tool 2.0 for randomized trials (RoB2) [15]. The five domains in RoB2 included the randomization process, deviations from intended interventions, missing outcome data, measurement of outcomes, and bias in selective reporting. The overall risk of bias was assessed using the Cochrane tool [14]. If there were any disagreements in assessing bias, two researchers (P-C, Y and Y-T, L) jointly discussed the findings to reach a consensus. A third researcher (C-C, L) was available to address any disagreements if a consensus could not be reached.

### Publication bias

To check for small-study effects, we reported publication bias by constructing funnel plots and funnel plot asymmetry, including Begg’s [16] and Egger’s [17] tests. For subgroup analysis with fewer than 10 studies, funnel plot asymmetry assessment is not applicable to avoid low power, according to the Handbook for Systematic Reviews of Interventions [18].

### Statistical methods

Since both our primary and secondary outcomes yielded dichotomous data, we calculated the risk ratios (RRs) with 95% confidence intervals (CIs) to determine the intervention effect. Intent-to-treat data were preferred where possible and available. Pooled estimates of effects were derived using a random-effects model because of the expected differences in patient characteristics across the included trials and the foreseeable complexity of GEM implementation. Heterogeneity was assessed using a chi-square test and considered significant when the p-value was less than 0.10 or the I^²^ was > 50%. If heterogeneity existed, we explored the individual trial characteristics to identify potential sources of heterogeneity using pre-planned subgroup analyses or leave-one-out sensitivity analyses. We used RevMan 5.4 software to analyze the data for the meta-analysis and construct the funnel plot. Significance tests for publication bias with Begg’s and Egger’s tests were performed using the “meta” package in the R software 4.2.1.

### Ethics

This study used open public available data. Ethical approval was exemplified from the Institute Review Board of the National Cheng Kung University Hospital.

## Results

### Selection of studies

**Fig. 1:**
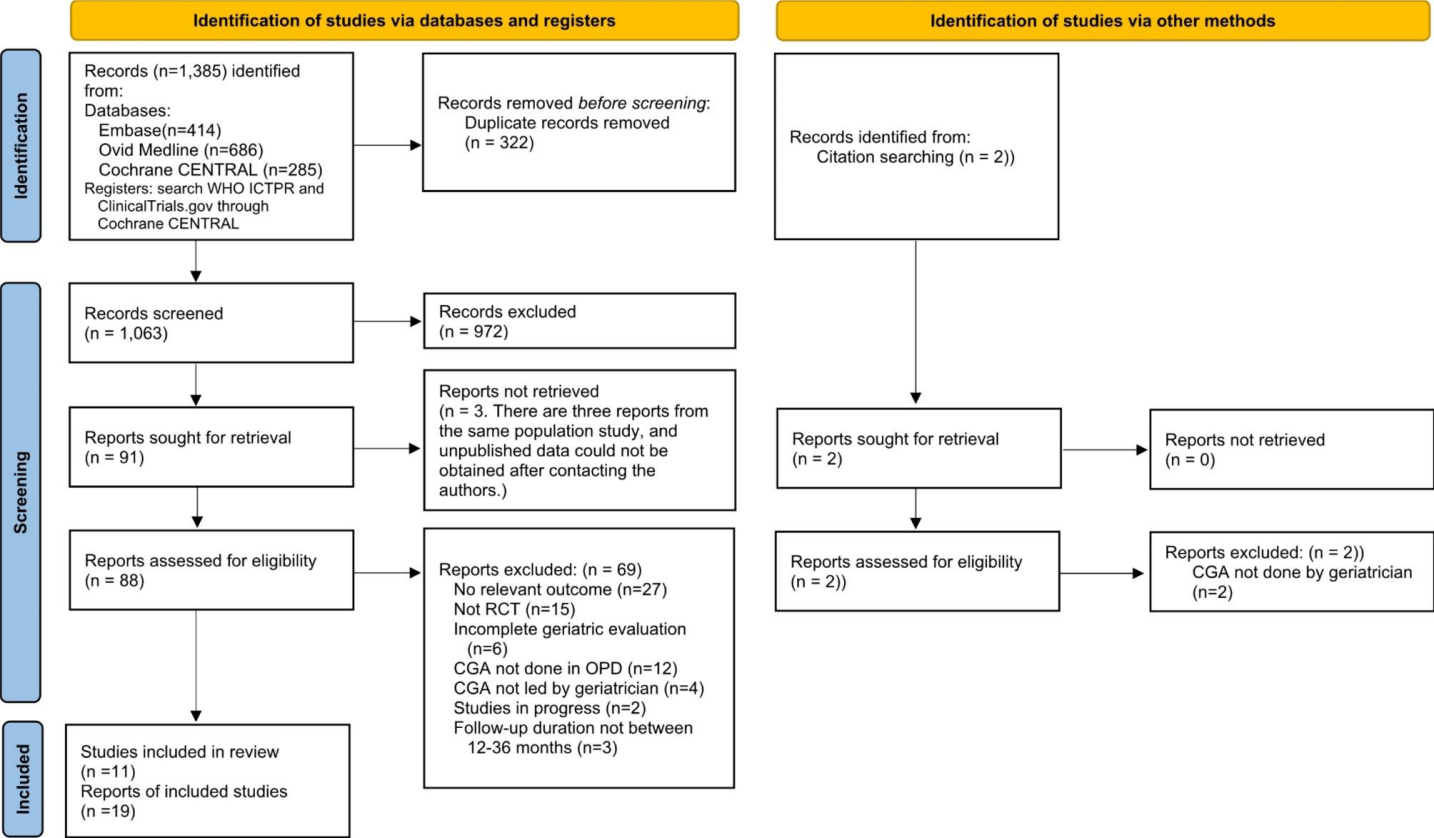
PRISMA 2020 flow diagram for studies selection

Studies were selected based on the PRISMA flowchart (Fig. 1). Of 1,385 records identified by the literature search from Embase, Ovid Medicine, and Cochrane CENTRAL, 19 reports were included in our review based on the inclusion and exclusion criteria. Since there were serial publications from the same population with different follow-up periods and outcome variables, we categorized these 19 reports into 11 studies to avoid repeated calculations. Four of the 19 reports were clinical registers, [19] protocols, [20] or abstracts [21, 22] without the availability of detailed outcome numbers. Consequently, 15 reports [11, 12, 23–35] from 11 studies were included in this meta-analysis.

### Characteristics of included studies

The characteristics of the total 11 included studies are summarized in Table 1. The included studies were published from 1987 to 2016, comprising 7,993 older participants, of which 3,571 were assigned to the GEM group and 4,422 to the usual care group. Among the 15 reports included in the meta-analysis, one was a cluster-RCT, [11] and 14 others were RCTs [12, 23–35]. Nine studies were conducted in the USA, [23–34] one in the United Kingdom, [11] and one in Sweden [12, 19–22, 35]. All studies were published in English.

**Table 1:** Eleven studies included

Study	Included Reports	Country	Patient Sources	Inclusion criteria	Patient Number*	Age (Mean)*	Male (%)*
Williams	Williams 1987	USA	Referrals to Geriatric ambulatory consultive services in a community hospital through an agency for older or chronically ill persons.	≥ 65 y/o community-dwelling adults with functional decline or 3 or more medication	58/59	76/77	36/44
Epstein	Epstein 1990	USA	A community health maintenance association	1. ≥74 y/o 2. 70–74 y/o with fair or worse health status rated by a primary physician or experiencing very likely or probable deterioration	185/205	76.7/76.9	49/52
Rubin	Rubin 1993	USA	Inpatient services from a single 900-bed acute-care county teaching hospital	≥ 70 y/o, indigent, acutely ill older patients during admission, who were both: 1. At high risk of hospital readmission for inpatient treatment to stabilize acute episodes of chronic illness 2. Good candidates for OPD management of existing chronic conditions as an alternative to inpatient treatment.	97/97	76.8/76.6	41/36
Silverman	Silverman 1995	USA	Four hospital-based ambulatory geriatric assessment clinics and community physicians’ offices	1. ≥65 y/o, had Medicare Part B or Medicaid; experiencing instability or had a change in their health status (risk for institutionalization or need intervention to deal with severe deterioration) 2. 60–65 with a clear need for care (few cases)	239/203	74.6/74.6	22/17
Toseland	Engelhardt 1996 Toseland 1997	USA	Outpatients from a single 450-bed Veterans affairs medical center	1.≥55 y/o with ≥ 10 outpatient visits in the previous 1 year 2. 55–75 y/o with at least 1 ADL + 2 IADL impairments 3. ≥75 y/o with 2 ADL or IADL impairments	80/80	72.6/71.7	100/100
Reuben	Reuben 1999	USA	Community-based sites	≥ 65 y/o community-dwelling adults with at least one impairment of four conditions (falls, urinary incontinence, depressive symptoms, or functional impairment)	180/183	75.8/75.9	27/20
Burns	Burns 1995 Burns 2000	USA	Any patient admitted to either the medical, surgical, or neurology services in a single Veterans affairs medical center	≥ 65 y/o with 2 of the following: (1) ≥ 1 ADL deficits (2) ≥ 2 chronic medical conditions (3) ≥ acute care hospitalizations in the previous 1 year (4) ≥ 6 prescription drugs	60/68	71.7/70.8	94/100
Boult	Boult 2001	USA	Ambulatory clinic in a community hospital	≥ 70 y/o community-dwelling with Medicare and high risk for hospital admission in the future	294/274	78.7/78.9	58/54
Fletcher	Fletcher 2004	UK	109 general practices from UK Medical Research Council General Practice Research Framework	≥ 75 y/o	1822/2733	81.6/81.3	36/37
Phibbs	Cohen 2002 Phibbs 2006	USA	Inpatients from 11 Veterans Affairs medical centers	≥ 65 y/o, hospitalized in a medical or surgical ward, with an expected length of stay of at least 2 days, and a frail condition	348/346	74.2 †	98†
Ekdahl (AGE-FIT trial)	Clinical trial 2011 Mazya 2013 (Protocol) Ekdahl 2014 (Abstract) Ekdahl 2015 (Abstract) Ekdahl 2015 Ekdahl 2016	Sweden	A population-based administrative database maintained by the county council	≥ 75 y/o community-dwelling, with ≥ 3 concomitant diagnoses and admitted to the hospital for inpatient care ≥ 3 times during the past year	208/174	82.3/82.7	53/50

*Presented as CGA/Control group; †Data only available for the entire study population (n = 1388); y/o: years old

There are two RCT with a 2 × 2 factorial design. Cohen et al. [33, 34] assigned participants to receive either care in a geriatric evaluation and management unit (GEMU) or usual inpatient care (UIPC), followed by either care at a geriatric evaluation and management clinic (GEMC) or usual outpatient care (UOPC). We derived outcomes from the UIPC-GEMC and UIPC-UOPC groups as we focused on outpatient GEM. In a large community-based study conducted by Fletcher et al., [11] all included participants who received a short questionnaire were allocated to universal or targeted assessment with subsequent management by the geriatric team versus the primary care team. In the universal group, an in-depth assessment was performed on all individuals, while in the targeted group, only participants with three or more problems in the short questionnaire received an in-depth assessment. The exact number of participants with detailed GEM in the targeted group remains unknown. Therefore, we included only the universal group with subsequent randomization to a geriatrician or primary care physician. In the RCT conducted by Epstein et al., [24] there was also a randomized group of “second opinion internists,” which we did not include in our meta-analysis. Our aim was to examine the effect of intervention from geriatricians other than internists.

Although all the studies had GEM performed in outpatient settings, the participants’ recruitment sources differed. Two studies were collected from administrative databases (Fletcher, Ekdahl), two from community-based sites (Reuben, Epstein), four from hospital-based outpatient clinics (Williams, Silverman, Toseland, Boult), and three from inpatient records (Rubin, Phibbs, Burns). Five of the studies (Williams, Epstein, Rubin, Toseland, Bruns, Boult) were single-center, and the remaining six were multi-centered.

The inclusion criteria were diverse among reports; however, all participants were older than 55-year-old with at least one of the following frail conditions: frailty, functional decline, increased utilization of health care, polypharmacy, multiple chronic medical conditions, high risk for future hospital admission, or current inpatient admission. One exception is the study by Fletcher et al., [11] which included the general population older than 75-year-old without mentioning certain conditions. Nursing-home residents were excluded in eight studies, [11, 12, 21–23, 25–29, 31–35] and patients with terminal illness were excluded in five studies [11, 25–27, 31, 33, 34]. The average age of both groups was 72–82 years. However, one study by Toseland et al. included younger participants above 55 years old [29]. The proportion of males in the included studies ranged from 20 to 100%. Males were the highest in number in three studies (Toseland, Bunrs, and Phibbs) performed in veterans’ hospitals. The follow-up period was between 12 and 36 months, with the majority being 12 months. Table S3 presents a list of outcomes in their cohort.

### Risk of bias

**Fig. 2:**
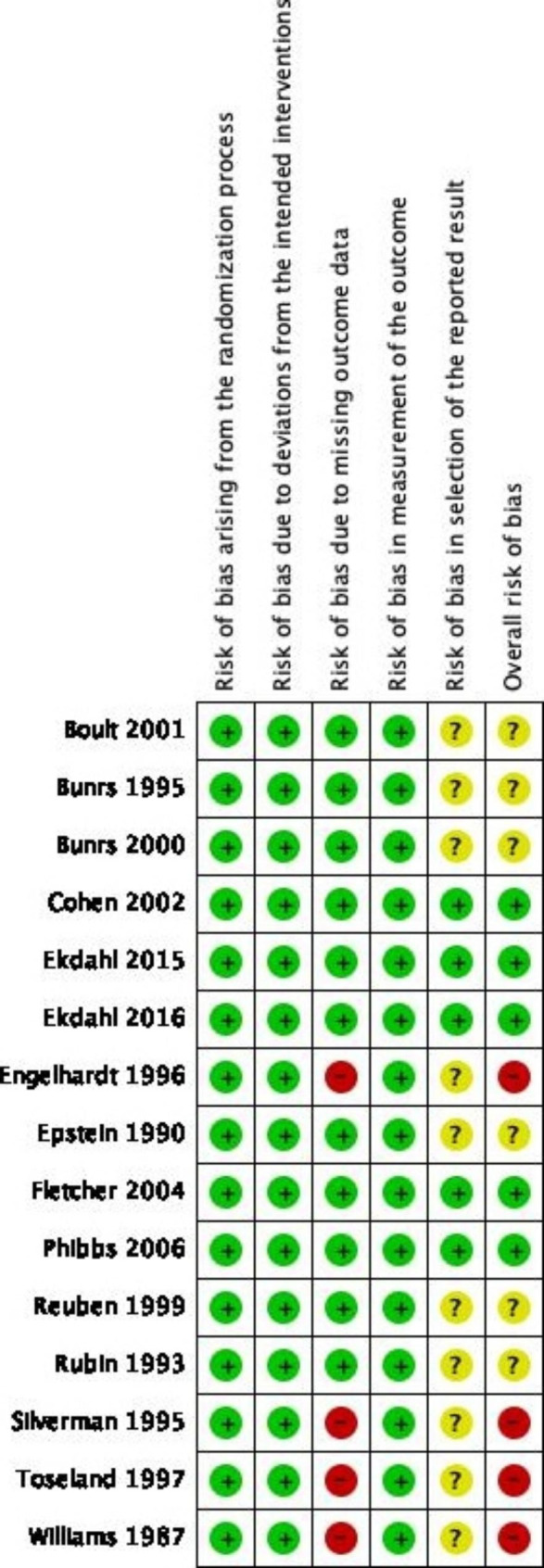
Summary of risk of bias of the included reports in the meta-analysis (n = 15)

Assessment of the risk of bias for the 15 included articles is shown in Fig. 2 and Figure S1. Four reports [23, 27–29] were rated as overall high risk, six [24–27, 30–32] rated as unclear risk, and five [11, 12, 33–35] as low risk. The four reports with overall high risk were from three studies with a high risk of bias due to missing outcome data. In a study by Williams, [23] 33 people were lost after randomization before the intervention started. Toseland and Engelhardt [28, 29] used intention-to-treat analysis in their studies; however, 7 and 9 participants in the GEM and conventional care groups, respectively, requested not to be interviewed by the 24-month assessment. In another report by Silverman, [27] the recruitment schedule was altered because of significantly higher attrition in the intervention group. It is likely that missing participants could affect the outcome. In the trials conducted by Rubin [25] and Epstein, [24] per-protocol analysis was used because ineligible patients were excluded after randomization. Nevertheless, there was a clear description that the missing data did not impact the results. Therefore, both reports were rated as low risk in the third risk of bias domain. Seven reports were rated as an unclear risk in selecting the reported result, as there were no available pre-published protocols.

### Results of meta-analysis

#### Primary outcome: mortality

**Fig. 3:**
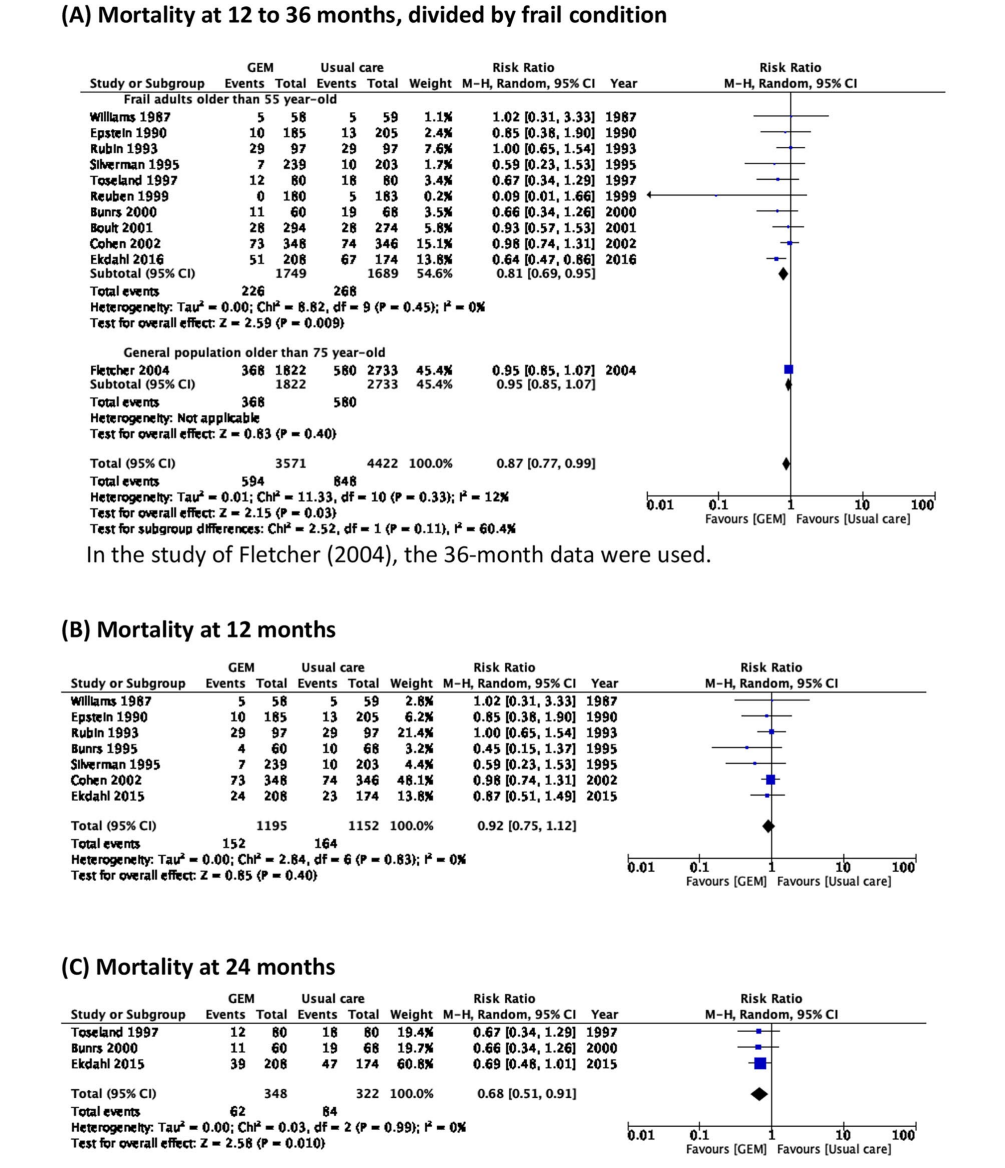
Mortality

Eleven reports were included to evaluate the impact of GEM on mortality [11, 12, 23–25, 27, 29–33]. While the population was the same with different follow-up periods, the outcome with the longest follow-up time was selected. The results of the meta-analysis indicated that there was a benefit of GEM intervention on mortality (RR = 0.87, 95% CI = 0.77–0.99), with low heterogeneity (p = 0.33, *I*^*2*^ = 12%) (Fig. 3.A). During the leave-one-out sensitivity test, the study by Fletcher et al., [11] which included all adults older than 75-year-old without certain frail conditions, the trend of benefit increased (RR = 0.81, 95% CI = 0.69 to 0.95), with a reduction in heterogeneity (p = 0.45, *I*^2^ = 0%). Additionally, no significant benefit was found after stratifying studies by mean age 70–75-year-old (RR = 0.86, 95% CI = 0.68–1.08, *I*^2^ = 0%), 75–80-year-old (RR = 0.93, 95% CI = 0.70–1.25, *I*^2^ = 0%), or over 80-year-old (RR = 0.80, 95% CI = 0.54–1.18, *I*^2^ = 83%) (Figure S2).

Subgroup analysis was performed according to the follow-up time (12 months, 15–18 months, 24, and 36 months). Follow-up durations of 12 months (Fig. 3.B) and 15–18 months (Figure S3) revealed a non-significant but beneficial trend toward the outpatient GEM group (12 months: RR = 0.92, 95% CI = 0.75–1.12; 15–18 months: RR = 0.96, 95% CI = 0.82–1.12) with low heterogenicity (p = 0.83, *I*^2^ = 0%; p = 0.44, *I*^2^ = 0%). Three reports [22, 29, 31] were included in the meta-analysis of the 24-month mortality. The GEM intervention was associated with significantly lower mortality (RR = 0.68, 95% CI = 0.51–0.91) with consistently low heterogeneity (p = 0.99, *I*^2^ = 0%) (Fig. 3.C). Only two trials [11, 12] reported 36-month mortality rates. The forest plot demonstrated no significant difference in mortality between the GEM and conventional care groups with high heterogeneity (RR = 0.80, 95% CI = 0.54–1.18; p = 0.02, *I*^2^ = 83%) (Figure S4).

Terminally ill patients were excluded in five [11, 21, 23, 27, 29] of the 11 studies. The subgroup analysis of these five studies showed no additional benefit of mortality in the GEM group (RR = 0.94, 95% CI = 0.85–1.05; I^2^ = 0%) (Figure S8). Conversely, in the subgroup analysis of six studies [12, 19, 25, 26] that did not exclude terminally ill patients, there is a 16% more risk reduction in mortality with outpatient GEM than usual care (RR = 0.71, 95% CI = 0.57–0.89, I^2^ = 0%) compared to the original pooled analysis (RR = 0.87, 95% CI = 0.77–0.99, I^2^ = 12%) (Figure S8).

### Secondary outcome: nursing-home admission

**Fig. 4:**
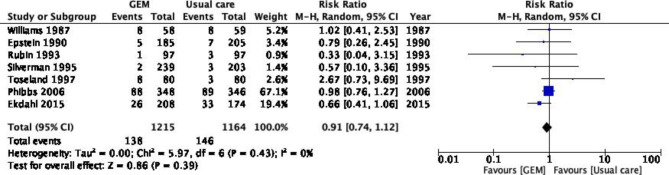
Number of patients with nursing-home admission (12–24 months)

A meta-analysis of seven trials [23–25, 27, 29, 34, 35] revealed a trend of non-significant effectiveness of outpatient GEM intervention in nursing-home admission during 12- to 24-month follow-up (RR = 0.91, 95% CI = 0.74–1.12). The heterogeneity was found to be low (p = 0.43, *I*^2^ = 0%) (Fig. 4). The result from Engelhardt et al. [28] with a 16-month follow-up was not included in the seven trials since it shared the same population as Toseland et al. [29] with a 24-month follow-up. Both subgroup analyses at 12 and 24 months did not show a significant impact of outpatient GEM over conventional care (Figure S5-S6). Ekdahl et al. [16] was the only report with a 36-month follow-up; therefore, the results were not integrated. In the subgroup analysis of studies that excluded terminally ill patients, both groups had a non-significant risk reduction in nursing home admissions (Figure S9).

### Publication bias

For the primary outcome of 12–36-month mortality, a funnel plot is shown in Figure S10. There is little evidence of small-study effects based on Egger’s (p = 0.0705) [17] and Begg’s tests (p = 0.1391). [16] Publication bias of other subgroups or secondary outcomes was not performed because the number of studies included was less than 10.

## Discussion

In this meta-analysis of 11 RCTs, we demonstrated a pooled effectiveness of 13% risk reduction on mortality with low heterogeneity in outpatient GEM compared to conventional care during the follow-up period from 12 to 36 months. Positive effectiveness was maintained and strengthened to 19% after excluding one study that included the general older population instead of the vulnerable older adults [11]. In addition, a survival benefit was found specifically in the 24-month subgroup analysis, with a 32% risk reduction. No significant impact was seen on nursing-home admission at 12–24 months of follow-up. To the best of our knowledge, this is the latest systematic review and meta-analysis since 2004^10^ on outpatient GEM to evaluate mortality and the first to explore nursing-home admission with specific inclusion criteria.

Our results of overall survival benefit in the outpatient GEM group differed from those of previous meta-analyses conducted by Kuo et al. in 2004, [10] Stuck et al. in 1993, [6] and Briggs et al. in 2022, [36] all of which did not reveal statistical differences in mortality. One possible explanation for the discrepancy between our study and the earlier two reviews [6, 10] could be that the newer, more well-designed RCTs in outpatient settings added to the evidence base were published in recent decades with longer follow-up periods, [11, 12, 35] along with a more thorough search strategy and attentive eligibility criteria to identify earlier studies [26, 28].

A recent Cochrane meta-analysis by Briggs et al. suggested that CGA caused little or no difference in mortality among community-dwelling, frail, and older adults during a median follow-up of 12 months [36]. However, Briggs et al. adopted a broader definition of CGA, given the community nature of the targeted population. For example, studies of home-based CGA were included in the analysis. Furthermore, CGA could be delivered by specialist nurses or therapists with gerontological expertise; however, the participation of geriatricians was not mentioned. These findings differed from our aim to explore the effectiveness of GEM led by geriatricians in outpatient settings. Furthermore, GEM performed in patients discharged from the hospital was excluded. In our opinion, these patients were at a high risk of vulnerability that could benefit from outpatient GEM.

Our results of survival benefit among subgroup analysis were seen at 24-month, but not at shorter interval of 12-month or 15-to 18-month follow-ups. From these findings, it can be noted that the advantages of outpatient GEM on decreased mortality may require a longer duration of continuous follow-up. In contrast, inpatient CGA showed benefits as early as six months, [6, 37, 38] with consistent mortality reduction after 12 months [39]. Meta-analysis of home-based CGA also revealed a reduction in mortality at the 6-month follow-up [38] and a positive pooled effect ranging from 3 to 36 months [8]. Our finding that 36-month mortality did not show a significant benefit in outpatient GEM should be interpreted with caution because there were only two reports with high heterogeneity (RR = 0.80, 95% CI = 0.54–1.18; I^2^ = 83%) and non-uniform inclusion criteria. One report included only frail patients, [12] while the other included the general population [11]. Future RCTs with longer duration of survival follow-up and more homogeneous targeted populations are warranted to confirm the hypothesized sustained long-term benefit in outpatient GEM.

The subgroup analysis including six studies [12, 19, 25, 26] that included terminally ill patients revealed a 16% reduced risk of 12-36-month mortality. On the other hand, our sub-group analysis for the five studies excluding terminally ill patients did not revealed the significant benefit on survival. Although these six [12, 19, 25, 26] did not describe the detailed number of terminal-ill patients, we believed the benefit of reduced mortality from outpatient GEM may not be limited to terminal-ill status.

Our finding that the non-significant effects of outpatient GEM on nursing-home admission between 12 and 24 months was consistent with a recent meta-analysis that included both outpatient and home CGA, with a focus on community-dwelling frail older adults [36]. A previous meta-analysis showed no impact on “living at home” at 12 and 24 months [6]. In contrast, inpatient CGA was proven beneficial in reducing nursing-home admission with a 3 to 12 months follow-up period in previous studies [6, 7, 39, 40]. Contentious benefits were found in home CGA, with one meta-analysis reporting a decrease in the long-term institutional facility admission rate [8] and a later study reporting no significant benefit [9]. The possible reason that outpatient GEM failed to provide benefits may be due to the limitation in tracking social or economic changes as well as compliance due to the nature of outpatient interventions instead of intensive follow-up of inpatient or home visits [10]. Although hospice or palliative care nursing home admissions may differ from “normal” nursing home admissions, the result of non-significant benefit remained regardless of the exclusion of terminal-ill patients.

Our study has several limitations. First, three of the included studies, responsible for six reports, [26, 28, 29, 31, 33, 34] were implemented in veterans’ hospitals with nearly 100% male participants. However, even after excluding these three studies, the pooled risk ratio for overall mortality in outpatient GEM over conventional care in frail older people was still significant (RR = 0.77, 95% CI = 0.62–0.95; *I*^2^ = 2%) (Figure S7). Second, generalization of the results was limited because all 11 included studies were from developed countries (the United States, the United Kingdom, and Sweden), even though we did not limit nationality in our search strategy. Third, our inclusion criterion for age was as young as 55 years. However, the frailty of younger patients may differ from that of older patients (> 75 years). Sensitivity tests were performed to evaluate how excluding the study that involved patients under the age of 65 would affect our findings on survival and nursing-home admission. If we leaved out the Toseland et al.‘s study [29] that included participants as low as 55-year-old, the additional survival benefit is still seen (RR = 0.88, 95% CI = 0.77–1.0, *I*^*2*^ = 15%) while the benefit of outpatient GEM on nursing-home admission remained non-significant (RR = 0.89, 95% CI = 0.72–1.10, *I*^2^ = 0%). Therefore, we concluded that the effect of this age discrepancy may be small. Fourth, the latest RCT identified in this systematic review was published in 2016, although our inception date was January 2022. This is due to insufficient RCTs, particularly in geriatric outpatient care. Fifth, palliative or terminally ill patients may have different characteristics from the general geriatric frail population, and there was a limited description in our included studies. Sixth, few qualified RCTs on outpatient GEM with follow-up period more than 24 months resulted in the apparent inter-study heterogeneity. Lastly, the risk of bias in selecting reported results was unclear in most of the included studies because there were no available protocols. This could also affect publication bias.

## Conclusions

In conclusion, this systematic review and meta-analysis confirmed that outpatient GEM benefited patient survival, not nursing-home admission. We believe that targeting frail older individuals significantly reduced mortality compared to targeting the general population. In the subgroup analysis, the effectiveness of outpatient GEM on mortality was disclosed only during the 24-month follow-up period. Future research on outpatient GEM involving a larger cohort is warranted to support our findings.

## Electronic supplementary material

Below is the link to the electronic supplementary material.

Supplementary Material 1

## Data Availability

The datasets generated during and/or analysed during the current study are available from the corresponding author on reasonable request.

## Abbreviations

GEM: Geriatric evaluation and management
RCTs: Randomized controlled trials
CGA: Comprehensive geriatric assessment
ADL: Activities of daily living
RRs: Risk ratios
CIs: Confidence intervals
GEMU: Geriatric evaluation and management unit
UIPC: Usual inpatient care
GEMC: Geriatric evaluation and management clinic
UOPC: Usual outpatient care

## References

[1] Guiding principles for the (2012). Care of older adults with multimorbidity: an approach for clinicians. Guiding principles for the care of older adults with multimorbidity: an approach for clinicians: american Geriatrics Society Expert Panel on the care of older adults with Multimorbidity. J Am Geriatr Soc.

[2] Parker SG, McCue P, Phelps K, McCleod A, Arora S, Nockels K (2018). What is comprehensive geriatric assessment (CGA)? An umbrella review. Age Ageing.

[3] Pilotto A, Cella A, Pilotto A, Daragjati J, Veronese N, Musacchio C (2017). Three decades of comprehensive geriatric assessment: evidence coming from different healthcare settings and specific clinical conditions. J Am Med Dir Assoc.

[4] O’Shaughnessy Í, Robinson K, O’Connor M, Conneely M, Ryan D, Steed F (2022). Effectiveness of acute geriatric unit care on functional decline, clinical and process outcomes among hospitalised older adults with acute medical complaints: a systematic review and meta-analysis. Age Ageing.

[5] Chen Z, Ding Z, Chen C, Sun Y, Jiang Y, Liu F (2021). Effectiveness of comprehensive geriatric assessment intervention on quality of life, caregiver burden and length of hospital stay: a systematic review and meta-analysis of randomised controlled trials. BMC Geriatr.

[6] Stuck AE, Siu AL, Wieland GD, Adams J, Rubenstein LZ (1993). Comprehensive geriatric assessment: a meta-analysis of controlled trials. Lancet.

[7] Ellis G, Gardner M, Tsiachristas A, Langhorne P, Burke O, Harwood RH (2017). Comprehensive geriatric assessment for older adults admitted to hospital. Cochrane Database Syst Rev.

[8] Elkan R, Kendrick D, Dewey M, Hewitt M, Robinson J, Blair M (2001). Effectiveness of home based support for older people: systematic review and meta-analysis. BMJ.

[9] Huss A, Stuck AE, Rubenstein LZ, Egger M, Clough-Gorr KM (2008). Multidimensional preventive home visit programs for community-dwelling older adults: a systematic review and meta-analysis of randomized controlled trials. J Gerontol A Biol Sci Med Sci.

[10] Kuo HK, Scandrett KG, Dave J, Mitchell SL (2004). The influence of outpatient comprehensive geriatric assessment on survival: a meta-analysis. Arch Gerontol Geriatr.

[11] Fletcher AE, Price GM, Ng ES, Stirling SL, Bulpitt CJ, Breeze E (2004). Population-based multidimensional assessment of older people in UK general practice: a cluster-randomised factorial trial. Lancet.

[12] Ekdahl AW, Alwin J, Eckerblad J, Husberg M, Jaarsma T, Mazya AL (2016). Long-term evaluation of the ambulatory geriatric assessment: a frailty intervention trial (AGe-FIT): clinical outcomes and total costs after 36 months. J Am Med Dir Assoc.

[13] Page MJ, McKenzie JE, Bossuyt PM, Boutron I, Hoffmann TC, Mulrow CD (2021). The PRISMA 2020 statement: an updated guideline for reporting systematic reviews. BMJ.

[14] Higgins JPT. In: Thomas J, Chandler J, Cumpston M, Li T, Page MJ, Welch VA, editors. Cochrane Handbook for systematic reviews of interventions version 6.3 (updated February 2022). Cochrane; 2022. https://training.cochrane.org/handbook. Accessed April 30, 2023.

[15] Sterne JAC, Savović J, Page MJ, Elbers RG, Blencowe NS, Boutron I (2019). RoB 2: a revised tool for assessing risk of bias in randomised trials. BMJ.

[16] Begg CB, Mazumdar M (1994). Operating characteristics of a rank correlation test for publication bias. Biometrics.

[17] Egger M, Davey Smith G, Schneider M, Minder C (1997). Bias in meta-analysis detected by a simple, graphical test. BMJ.

[18] Page MJ, Higgins JPT, Sterne JAC. Chapter 13: Assessing risk of bias due to missing results in a synthesis. In: Higgins JPT, Thomas J, Chandler J, Cumpston M, Li T, Page MJ, Welch VA, editors. Cochrane Handbook for Systematic Reviews of Interventions version 6.3 (updated February 2022). Cochrane, 2022. https://training.cochrane.org/handbook. Accessed April 30, 2023.

[19] Ekdahl A. Ambulatory geriatric evaluation - frailty intervention trial (AGE-FIT). https://clinicaltrials.gov/ct2/show/NCT01446757. Accessed October 18, 2022.

[20] Mazya AL, Eckerblad J, Jaarsma T, Hellström I, Krevers B, Milberg A (2013). The Ambulatory Geriatric Assessment - A Frailty intervention trial (AGe-FIT) - a randomised controlled trial aimed to prevent hospital readmissions and functional deterioration in high risk older adults: a study protocol. Eur Geriatr Med.

[21] Ekdahl AW, Wirehn AB, Jaarsma T, Unosson M, Alwin J, Husberg M (2014). Caring for elderly with multimorbidity: evaluation of ambulatory geriatric unit (AGU) (the AGe-FIT-study) - a randomized controlled trial. Eur Geriatr Med.

[22] Ekdahl AW, Wirehn AB, Alwin J (2015). Long-term evaluation of the ambulatory geriatric assessment-a frailty intervention trial (AGE-FIT)-clinical outcomes and total costs after 36 months. Eur Geriatr Med.

[23] Williams ME, Williams TF, Zimmer JG, Hall WJ, Podgorski CA (1987). How does the team approach to outpatient geriatric evaluation compare with traditional care: a report of a randomized controlled trial. J Am Geriatr Soc.

[24] Epstein AM, Hall JA, Fretwell M, Feldstein M, DeCiantis ML, Tognetti J (1990). Consultative geriatric assessment for ambulatory patients. A randomized trial in a health maintenance organization. JAMA.

[25] Rubin CD, Sizemore MT, Loftis PA, de Mola NL (1993). A randomized, controlled trial of outpatient geriatric evaluation and management in a large public hospital. J Am Geriatr Soc.

[26] Burns R, Nichols LO, Graney MJ, Cloar FT (1995). Impact of continued geriatric outpatient management on health outcomes of older veterans. Arch Intern Med.

[27] Silverman M, Musa D, Martin DC, Lave JR, Adams J, Ricci EM (1995). Evaluation of outpatient geriatric assessment: a randomized multi-site trial. J Am Geriatr Soc.

[28] Engelhardt JB, Toseland RW, O’Donnell JC, Richie JT, Jue D, Banks S (1996). The effectiveness and efficiency of outpatient geriatric evaluation and management. J Am Geriatr Soc.

[29] Toseland RW, O’Donnell JC, Engelhardt JB, Richie J, Jue D, Banks SM (1997). Outpatient geriatric evaluation and management: is there an investment effect?. Gerontologist.

[30] Reuben DB, Frank JC, Hirsch SH, McGuigan KA, Maly RC (1999). A randomized clinical trial of outpatient comprehensive geriatric assessment coupled with an intervention to increase adherence to recommendations see comments. J Am Geriatr Soc.

[31] Burns R, Nichols LO, Martindale-Adams J, Graney MJ (2000). Interdisciplinary geriatric primary care evaluation and management: two- year outcomes. J Am Geriatr Soc.

[32] Boult C, Boult LB, Morishita L, Dowd B, Kane RL, Urdangarin CF (2001). A randomized clinical trial of outpatient geriatric evaluation and management. J Am Geriatr Soc.

[33] Cohen HJ, Feussner JR, Weinberger M, Carnes M, Hamdy RC, Hsieh F (2002). A controlled trial of inpatient and outpatient geriatric evaluation and management. N Engl J Med.

[34] Phibbs CS, Holty JE, Goldstein MK, Garber AM, Wang Y, Feussner JR (2006). The effect of geriatrics evaluation and management on nursing home use and health care costs: results from a randomized trial. Med Care.

[35] Ekdahl AW, Wirehn AB, Alwin J, Jaarsma T, Unosson M, Husberg M (2015). Costs and Effects of an ambulatory geriatric unit (the AGe-FIT study): a randomized controlled trial. J Am Med Dir Assoc.

[36] Briggs R, McDonough A, Ellis G, Bennett K, O’Neill D, Robinson D (2022). Comprehensive Geriatric Assessment for community-dwelling, high-risk, frail, older people. Cochrane Database Syst Rev.

[37] Deschodt M, Flamaing J, Haentjens P, Boonen S, Milisen K (2013). Impact of geriatric consultation teams on clinical outcome in acute hospitals: a systematic review and meta-analysis. BMC Med.

[38] Rubenstein LZ, Stuck AE, Siu AL, Wieland D (1991). Impacts of geriatric evaluation and management programs on defined outcomes: overview of the evidence. J Am Geriatr Soc.

[39] Bachmann S, Finger C, Huss A, Egger M, Stuck AE, Clough-Gorr KM (2010). Inpatient rehabilitation specifically designed for geriatric patients: systematic review and meta-analysis of randomised controlled trials. BMJ.

[40] Van Craen K, Braes T, Wellens N, Denhaerynck K, Flamaing J, Moons P (2010). The effectiveness of inpatient geriatric evaluation and management units: a systematic review and meta-analysis. J Am Geriatr Soc.

