# Supplementary material for: Effectiveness of outpatient geriatric evaluation and management intervention on survival and nursing home admission: a systematic review and meta-analysis of randomized controlled trials
Source: BMC Geriatr. 2023 Jul 7;23:414. doi: 10.1186/s12877-023-04036-4 (PMC10329350; doi:10.1186/s12877-023-04036-4)

**Title:**

Effectiveness of outpatient geriatric evaluation and management intervention on survival and nursing home admission: a systematic review and meta-analysis of randomized controlled trials

Supplementary Data

[Table S1. PRISMA Checklist 2](#_Toc134173204)

[Table S2: Search Strategy 6](#_Toc134173205)

[Table S3. Lists of outcomes for included reports in the meta-analysis (n=15) 10](#_Toc134173206)

[Figure S1. Summary of risk of Bias of included reports in the meta-analysis (n=15) 11](#_Toc134173207)

[Figure S2. Overall mortality at 12-36 months divided by mean age 11](#_Toc134173208)

[Figure S3. Mortality at 15–18 months 11](#_Toc134173209)

[Figure S4. Mortality at 36 months 12](#_Toc134173210)

[Figure S5. Number of patients admitted to the nursing home (12 months) 12](#_Toc134173211)

[Figure S6. Number of patients admitted to the nursing home (24 months) 12](#_Toc134173212)

[Figure S7. Overall mortality at 12–36 months in frail older adults≥55 years old (excluding 3 studies done in Veterans’ hospital) 12](#_Toc134173213)

[Figure S8. 12-36M Mortality, stratified by exclusion of terminal-ill patients or not 13](#_Toc134173214)

[Figure S9. 12-24M Nursing home admission, stratified by exclusion of terminal-ill patients or not 13](#_Toc134173215)

[Figure S10. Funnel Plot of primary outcome (Mortality, 12-36 months) 14](#_Toc134173216)

# Table S1. PRISMA Checklist

| **Section and Topic** | **Item #** | **Checklist item** | **Location where item is reported** |
| --- | --- | --- | --- |
| **TITLE** | | |  |
| Title | 1 | Identify the report as a systematic review. | Title page 1-2 |
| **ABSTRACT** | | |  |
| Abstract | 2 | See the PRISMA 2020 for Abstracts checklist. | Page 1-2 |
| **INTRODUCTION** | | |  |
| Rationale | 3 | Describe the rationale for the review in the context of existing knowledge. | Page 3 |
| Objectives | 4 | Provide an explicit statement of the objective(s) or question(s) the review addresses. | Page 4-5 |
| **METHODS** | | |  |
| Eligibility criteria | 5 | Specify the inclusion and exclusion criteria for the review and how studies were grouped for the syntheses. | Page 7-8 |
| Information sources | 6 | Specify all databases, registers, websites, organisations, reference lists and other sources searched or consulted to identify studies. Specify the date when each source was last searched or consulted. | Page 6 |
| Search strategy | 7 | Present the full search strategies for all databases, registers and websites, including any filters and limits used. | Table S2 |
| Selection process | 8 | Specify the methods used to decide whether a study met the inclusion criteria of the review, including how many reviewers screened each record and each report retrieved, whether they worked independently, and if applicable, details of automation tools used in the process. | Page 8-9 |
| Data collection process | 9 | Specify the methods used to collect data from reports, including how many reviewers collected data from each report, whether they worked independently, any processes for obtaining or confirming data from study investigators, and if applicable, details of automation tools used in the process. | Page 8-9 |
| Data items | 10a | List and define all outcomes for which data were sought. Specify whether all results that were compatible with each outcome domain in each study were sought (e.g. for all measures, time points, analyses), and if not, the methods used to decide which results to collect. | Page 8-9 |
|  | 10b | List and define all other variables for which data were sought (e.g. participant and intervention characteristics, funding sources). Describe any assumptions made about any missing or unclear information. | Page 8-9 |
| Study risk of bias assessment | 11 | Specify the methods used to assess risk of bias in the included studies, including details of the tool(s) used, how many reviewers assessed each study and whether they worked independently, and if applicable, details of automation tools used in the process. | Page 9-10 |
| Effect measures | 12 | Specify for each outcome the effect measure(s) (e.g. risk ratio, mean difference) used in the synthesis or presentation of results. | Page 10-11 |
| Synthesis methods | 13a | Describe the processes used to decide which studies were eligible for each synthesis (e.g. tabulating the study intervention characteristics and comparing against the planned groups for each synthesis (item #5)). | Page 10-11 |
|  | 13b | Describe any methods required to prepare the data for presentation or synthesis, such as handling of missing summary statistics, or data conversions. | Page 10-11 |
|  | 13c | Describe any methods used to tabulate or visually display results of individual studies and syntheses. | Page10-11 |
|  | 13d | Describe any methods used to synthesize results and provide a rationale for the choice(s). If meta-analysis was performed, describe the model(s), method(s) to identify the presence and extent of statistical heterogeneity, and software package(s) used. | Page 10-11 |
|  | 13e | Describe any methods used to explore possible causes of heterogeneity among study results (e.g. subgroup analysis, meta-regression). | Page 10-11 |
|  | 13f | Describe any sensitivity analyses conducted to assess robustness of the synthesized results. | Page 10-11 |
| Reporting bias assessment | 14 | Describe any methods used to assess risk of bias due to missing results in a synthesis (arising from reporting biases). | Page 9-10 |
| Certainty assessment | 15 | Describe any methods used to assess certainty (or confidence) in the body of evidence for an outcome. | Not applicable |
| **RESULTS** | | |  |
| Study selection | 16a | Describe the results of the search and selection process, from the number of records identified in the search to the number of studies included in the review, ideally using a flow diagram. | Page 12  Figure 1. |
|  | 16b | Cite studies that might appear to meet the inclusion criteria, but which were excluded, and explain why they were excluded. | Page 12  Figure 1 |
| Study characteristics | 17 | Cite each included study and present its characteristics. | Page 12-15  Table 1 |
| Risk of bias in studies | 18 | Present assessments of risk of bias for each included study. | Page 15-16  Figure 2  Figure S1  Figure S10 |
| Results of individual studies | 19 | For all outcomes, present, for each study: (a) summary statistics for each group (where appropriate) and (b) an effect estimate and its precision (e.g. confidence/credible interval), ideally using structured tables or plots. | Figure 3, Figure 4, Figure S2-S9 |
| Results of syntheses | 20a | For each synthesis, briefly summarise the characteristics and risk of bias among contributing studies. | Page 16-18 |
|  | 20b | Present results of all statistical syntheses conducted. If meta-analysis was done, present for each the summary estimate and its precision (e.g. confidence/credible interval) and measures of statistical heterogeneity. If comparing groups, describe the direction of the effect. | Page 16-18  Figure 3, Figure 4, Figure S2-S9 |
|  | 20c | Present results of all investigations of possible causes of heterogeneity among study results. | Page 16-18 |
|  | 20d | Present results of all sensitivity analyses conducted to assess the robustness of the synthesized results. | Page 16-18 |
| Reporting biases | 21 | Present assessments of risk of bias due to missing results (arising from reporting biases) for each synthesis assessed. | Page 18  Figure S10 |
| Certainty of evidence | 22 | Present assessments of certainty (or confidence) in the body of evidence for each outcome assessed. | Not applicable |
| **DISCUSSION** | | |  |
| Discussion | 23a | Provide a general interpretation of the results in the context of other evidence. | Page 19 |
|  | 23b | Discuss any limitations of the evidence included in the review. | Page 22-24 |
|  | 23c | Discuss any limitations of the review processes used. | Page 22-24 |
|  | 23d | Discuss implications of the results for practice, policy, and future research. | Page 19-24 |
| **OTHER INFORMATION** | | |  |
| Registration and protocol | 24a | Provide registration information for the review, including register name and registration number, or state that the review was not registered. | Page 6 |
|  | 24b | Indicate where the review protocol can be accessed, or state that a protocol was not prepared. | Page 6 |
|  | 24c | Describe and explain any amendments to information provided at registration or in the protocol. | Not applicable |
| Support | 25 | Describe sources of financial or non-financial support for the review, and the role of the funders or sponsors in the review. | Page 25 |
| Competing interests | 26 | Declare any competing interests of review authors. | Page 25 |
| Availability of data, code and other materials | 27 | Report which of the following are publicly available and where they can be found: template data collection forms; data extracted from included studies; data used for all analyses; analytic code; any other materials used in the review. | Page 25 |

*From:*  Page MJ, McKenzie JE, Bossuyt PM, Boutron I, Hoffmann TC, Mulrow CD, et al. The PRISMA 2020 statement: an updated guideline for reporting systematic reviews. BMJ 2021;372:n71. doi: 10.1136/bmj.n71

For more information, visit: <http://www.prisma-statement.org/>

**Table S2: Search Strategy**

| **Database** | **#** | **Search syntax** | **Result** |
| --- | --- | --- | --- |
| **Embase.com (Elsevier)** | 1 | (aged OR aging  OR elder* OR geriatric* OR gerontolog* OR senior* OR senium* OR "old age*" OR senescen* OR (older NEAR/2 (adult* OR person* OR people OR patient* OR outpatient* OR population* OR men OR women OR male OR female OR subject* OR citizen*))):ti,ab,kw,de | 5,908,844 |
|  | 2 | "Aged"/exp OR "Elderly care"/exp OR "Geriatrics"/exp OR "gerontology"/exp OR "senescence"/exp | 3,526,555 |
|  | 3 | (outpatient* OR out-patient* OR clinics OR clinic OR outward* OR "out ward*" OR "office visit*" OR (ambulatory NEAR/3 (care OR setting* OR unit* OR center* OR patient* OR service*))):ti,ab,kw,de | 960,928 |
|  | 4 | "outpatient"/exp OR "ambulatory care"/exp OR "outpatient department"/exp OR "outpatient care"/exp | 304,249 |
|  | 5 | ((geriatric* NEAR/2 (assessment* OR evaluation* OR management OR comprehensive*)) OR CGA):ti,ab,kw,de | 29,501 |
|  | 6 | "Geriatric assessment"/exp | 18,856 |
|  | 7 | (#1 OR #2) AND (#3 OR #4) AND (#5 OR #6) AND [embase]/lim | **All: 1,743** |
|  | 8 | #7 AND ("randomized controlled trial"/de or "controlled clinical trial"/de or "randomization"/de or "intermethod comparison"/de or "double blind procedure"/de or "human experiment"/de OR (random* or placebo or assigned or allocated or volunteer or volunteers or (open NEXT/1 label) or ((double or single or doubly or singly) NEXT/1 (blind or blinded or blindly)) or "parallel group?" or crossover or "cross over" or ((assign* or match or matched or allocation) NEAR/5 (alternate or group? or intervention? or patient? or subject? or participant?)) OR (controlled NEAR/7 (study or design or trial))):ti,ab OR (compare or compared or comparison or trial):ti OR ((evaluated or evaluate or evaluating or assessed or assess) and (compare or compared or comparing or comparison)):ab) NOT (((random* NEXT/1 sampl* NEAR/7 ("cross section*" or questionnaire? or survey* or database?)):ti,ab not ("comparative study"/de or "controlled study"/de or "randomi?ed controlled":ti,ab or "randomly assigned":ti,ab)) OR ("Cross-sectional study"/de not ("randomized controlled trial"/de or "controlled clinical study"/de or "controlled study"/de or randomi?ed controlled:ti,ab or "control group?":ti,ab)) OR ((((case NEXT/1 control*) and random*) not randomi?ed controlled):ti,ab) OR ("Systematic review" not (trial or study)):ti OR (nonrandom* not random*):ti,ab OR "Random field*":ti,ab OR ("random cluster" NEAR/3 sampl*):ti,ab OR ((review:ab and review/it) not trial:ti) OR ("we searched":ab and (review:ti or review/it)) OR "update review":ab OR (databases NEAR/4 searched):ab OR ((rat or rats or mouse or mice or swine or porcine or murine or sheep or lambs or pigs or piglets or rabbit or rabbits or cat or cats or dog or dogs or cattle or bovine or monkey or monkeys or trout or marmoset?):ti and "animal experiment"/de) OR ("animal experiment"/de not ("human experiment"/de or "human"/de)))  Filter Source: Box 3.e, [Technical Supplement to Chapter 4: Searching for and Selecting Studies](https://training.cochrane.org/handbook/version-6/chapter-4-tech-suppl). Cochrane Handbook for Systematic Reviews of Interventions Version 6. (Syntax Translated from Ovid Embase to Elsevier Embase.com.) | **RCT: 414** |
| **MEDLINE (Ovid)** and Epub Ahead of Print, In-Process, In-Data-Review & Other Non-Indexed Citations | 1 | (aged OR aging  OR elder* OR geriatric* OR gerontolog* OR senior* OR senium* OR "old age*"  OR Senescence OR (older ADJ2 (adult* OR person* OR people OR patient* OR outpatient* OR population* OR men OR women OR male OR female OR subject* OR citizen*))).mp | 6,151,062 |
|  | 2 | exp "Aged"/ OR exp "Aging"/ OR exp "Healthy aging"/ OR exp "Geriatrics"/ | 3,544,981 |
|  | 3 | (outpatient* OR out-patient* OR clinics OR clinic OR outward* OR "out ward*" OR "office visit*" OR (ambulatory ADJ3 (care OR setting* OR unit* OR center* OR patient* OR service*))).mp | 594,504 |
|  | 4 | exp "Outpatients"/ OR exp "Ambulatory Care"/ OR exp "Ambulatory Care Facilities"/ | 125,810 |
|  | 5 | ((geriatric* ADJ2 (assessment* OR evaluation* OR management OR comprehensive*)) OR CGA).mp | 37680 |
|  | 6 | exp "Geriatric Assessment"/ | 30,645 |
|  | 7 | (1 OR 2) AND (3 OR 4) AND (5 OR 6) | **All:2501** |
|  | 8 | 7 AND (randomized controlled trial.pt. or controlled clinical trial.pt. or randomi*ed.ab. or placebo.ab. or drug therapy.fs. or randomly.ab. or trial.ab. or groups.ab. not (exp animals/ not humans.sh.))  Source: modified from Cochrane, <https://www.cochrane.org/MR000041/METHOD_search-strategies-identify-observational-studies-medline-and-embase> | **RCT: 686** |
| **Cochrane Library** | 1 | (aged OR aging OR elder* OR geriatric* OR gerontolog* OR senior* OR senium* OR "old age*" OR Senescence OR (older NEAR/1 (adult* OR person* OR people OR patient* OR outpatient* OR population* OR men OR women OR male OR female OR subject* OR citizen*))):ti,ab,kw | 578,109 |
|  | 2 | [mh "Aged"] OR [mh "Aging"] OR [mh "Healthy aging"] OR [mh "Geriatrics"] | 218,579 |
|  | 3 | (outpatient* OR out-patient* OR clinics OR clinic OR outward* OR "out ward*" OR "office visit*" OR (ambulatory NEAR/2 (care OR setting* OR unit* OR center* OR patient* OR service*))):ti,ab,kw | 97,420 |
|  | 4 | [mh "Outpatients"] OR [mh "Ambulatory Care"] Source: modified from Cochrane, <https://www.cochrane.org/MR000041/METHOD_search-strategies-identify-observational-studies-medline-and-embase>OR [mh "Ambulatory Care Facilities"] | 6,713 |
|  | 5 | ((geriatric* NEAR/1 (assessment* OR evaluation* OR management OR comprehensive*)) OR CGA):ti,ab,kw | 2775 |
|  | 6 | [mh "Geriatric assessment"] | 1,564 |
|  | 7 | (#1 OR #2) AND (#3 OR #4) AND (#5 OR #6) | **All:286** |
|  | 8 | #7 Limits: in Cochrane Reviews, Cochrane Protocols, Trials | **RCT:285** |


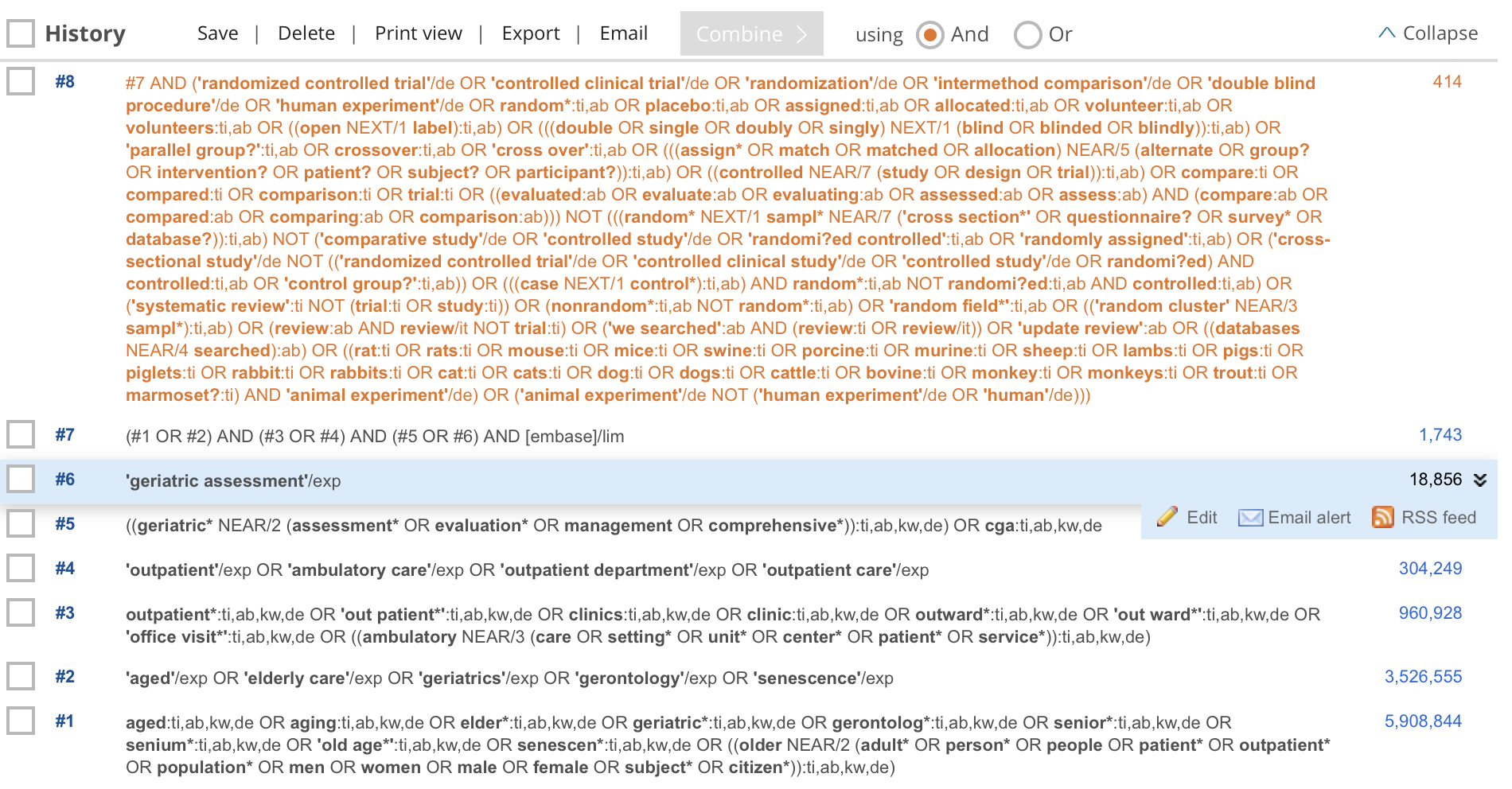


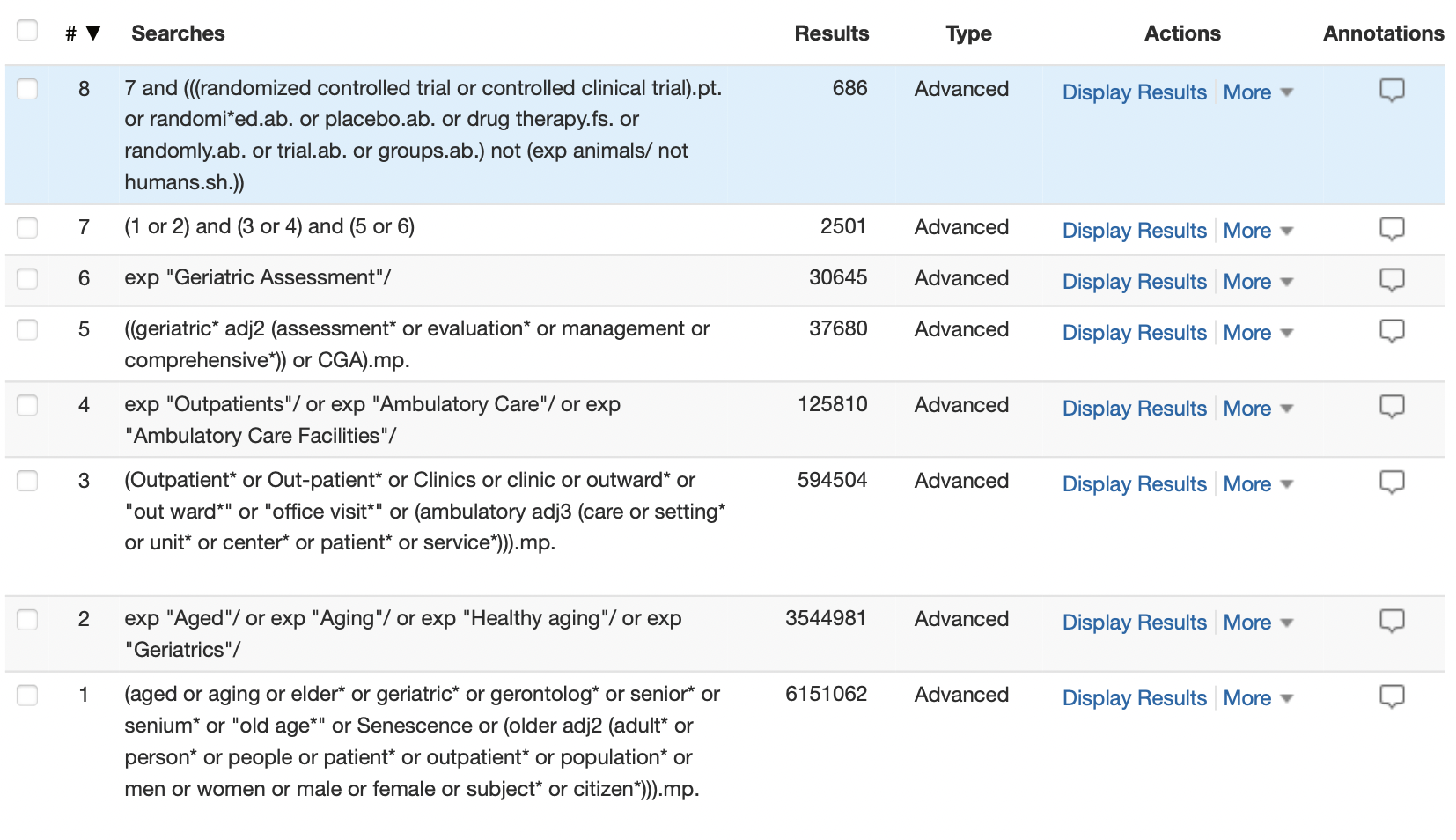


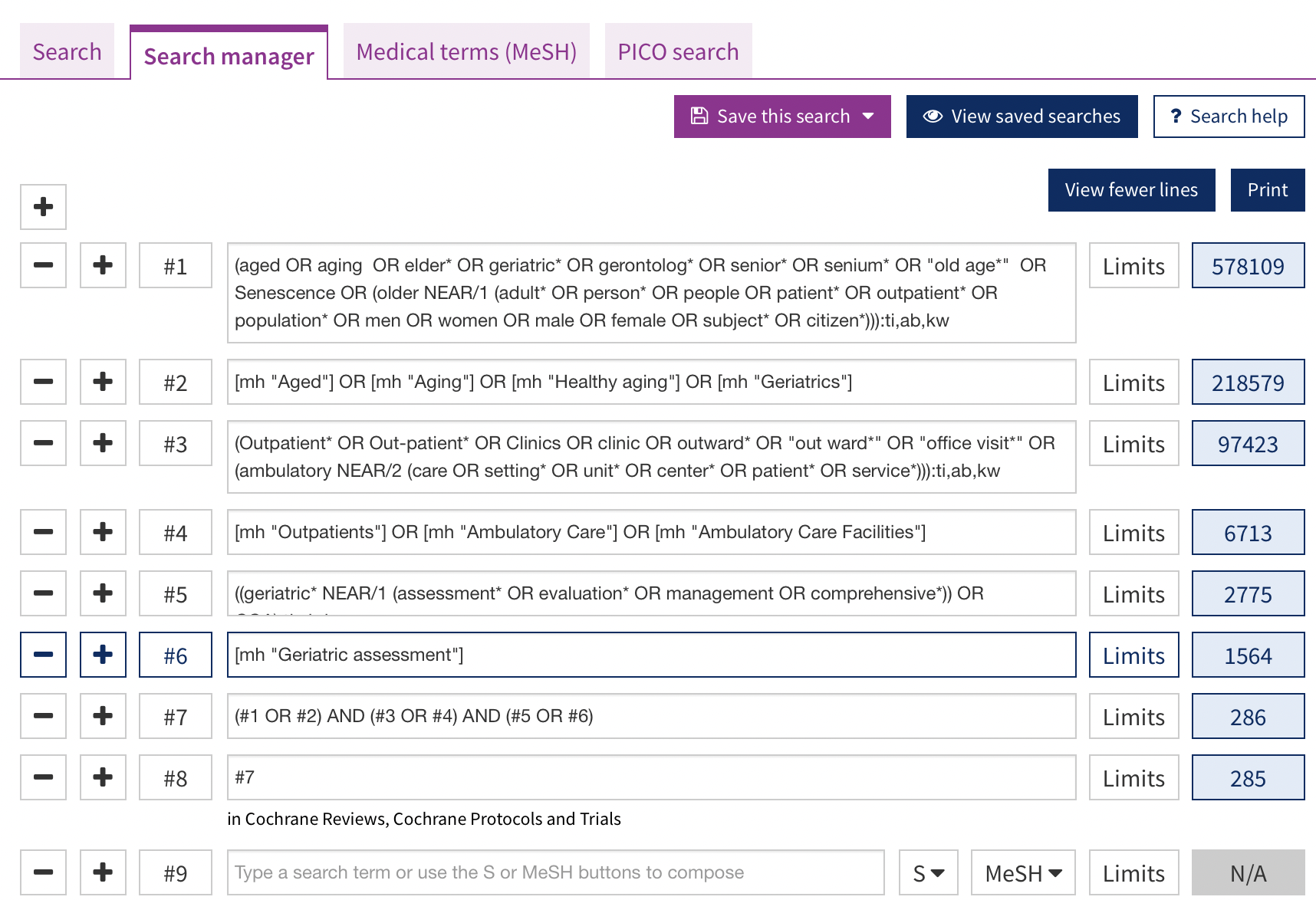


# Table S3. Lists of outcomes for included reports in the meta-analysis (n=15)

| **Reports** | **Outcome** | |
| --- | --- | --- |
|  | **Mortality** | **Nursing home admission** |
| Williams 1987 | 12-month | 12-month |
| Epstein 1990 | 12-month | 12-month |
| Rubin 1993 | 12-month | 12-month |
| Silverman 1995 | 12-month | 12-month |
| Engelhardt 1996^*^ | 16-month | 16-month |
| Toseland 1997^*^ | 24-month | 24-month |
| Reuben 1999 | 15-month | ------ |
| Burns 1995 † | 12-month | ------ |
| Burns 2000 † | 24-month | ------ |
| Boult 2001 | 18-month | ------ |
| Fletcher 2004 | 18-month, 36-month | ------ |
| Cohen 2002 § | 12-month | ------ |
| Phibbs 2006 § | ------- | 12-month |
| Ekdahl 2015 \|\| | 12-month; 24-month | 24-month |
| Ekdahl 2016 \|\| | 36-month | 36-month |

^*^ Same population under one study; †Same population under one study; § Same population under one study; || Same population under one study; ----- outcome not presented

# Figure S1. Summary of risk of Bias of included reports in the meta-analysis (n=15)


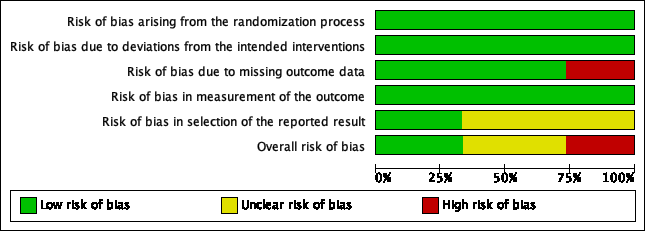


# Figure S2. Overall mortality at 12-36 months divided by mean age


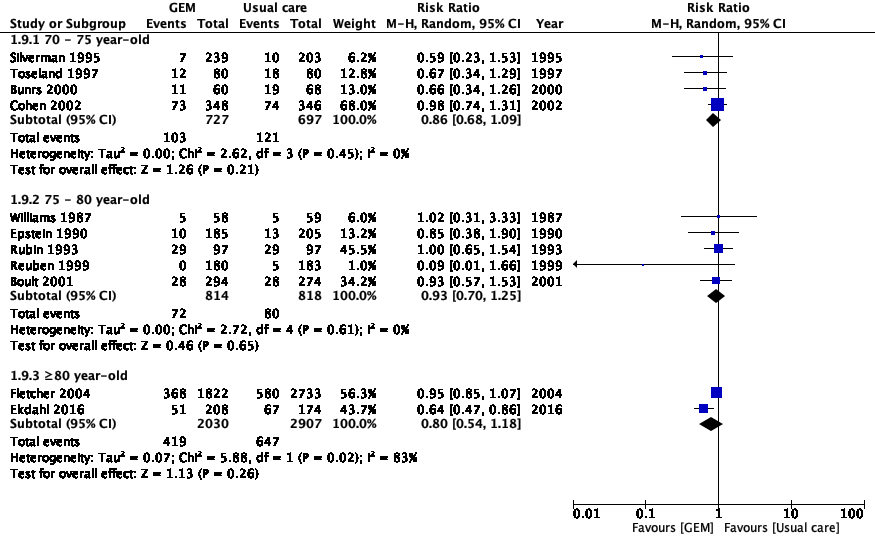


# Figure S3. Mortality at 15–18 months


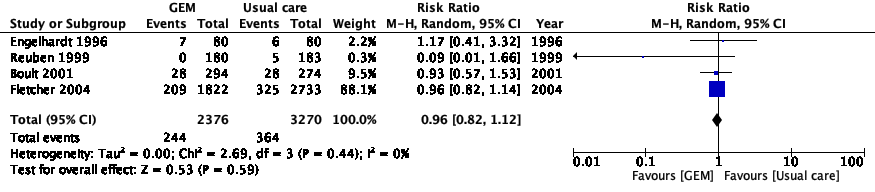


Engelhardt 1996(16-month), Reuben 1999 (15-month), Boult 2001 (18-month), Fletcher 2004 (18-month)

# Figure S4. Mortality at 36 months


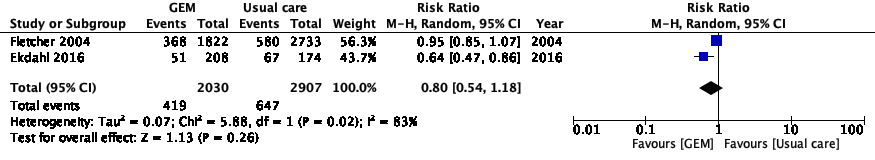


# Figure S5. Number of patients admitted to the nursing home (12 months)


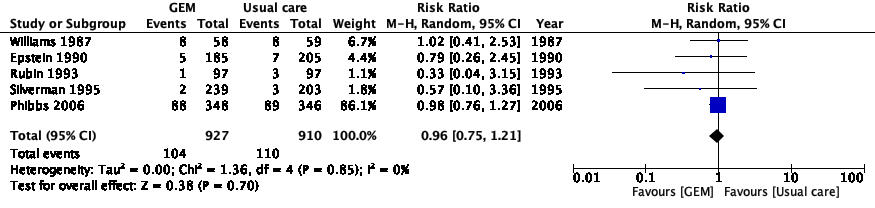


# Figure S6. Number of patients admitted to the nursing home (24 months)


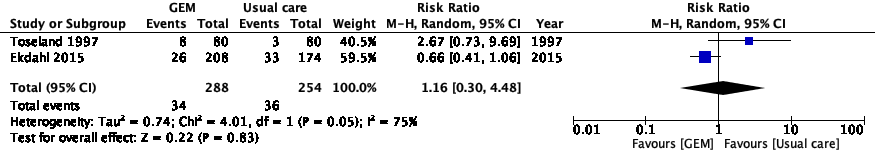


# Figure S7. Overall mortality at 12–36 months in frail older adults≥55 years old (excluding 3 studies done in Veterans’ hospital)


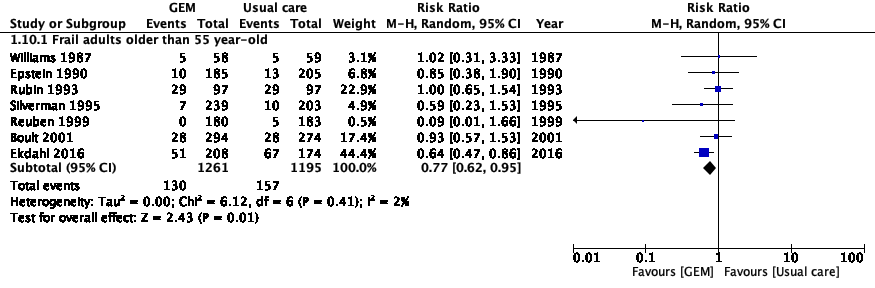


# Figure S8. 12-36M Mortality, stratified by exclusion of terminal-ill patients or not


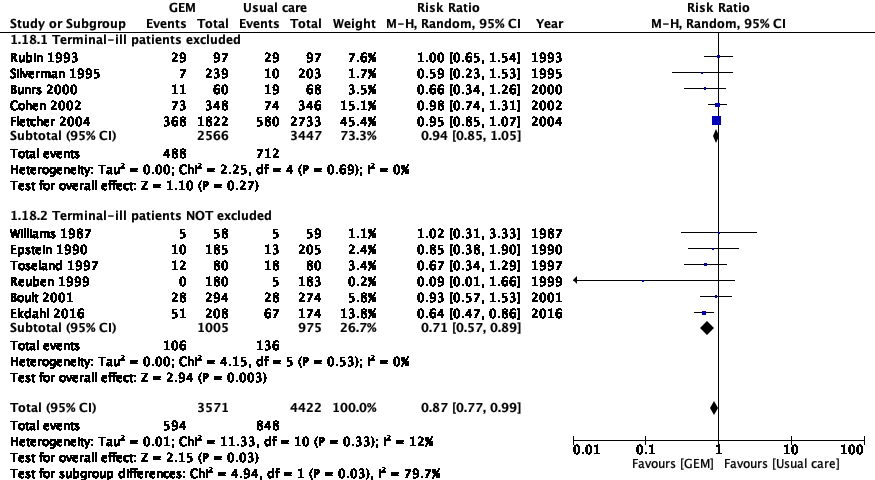


# Figure S9. 12-24M Nursing home admission, stratified by exclusion of terminal-ill patients or not


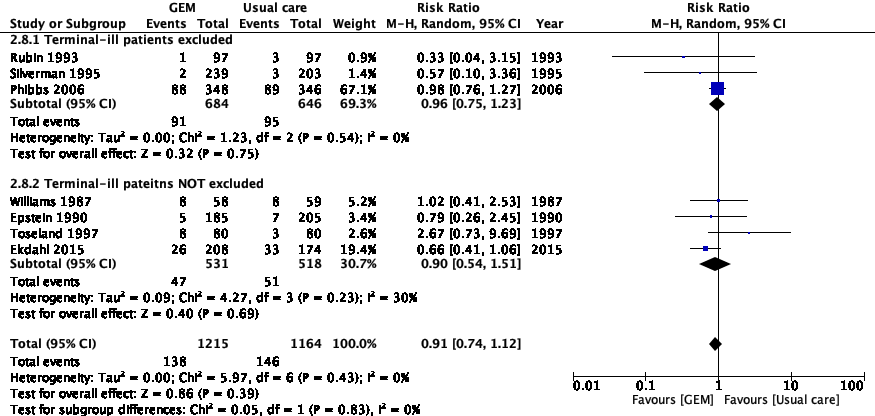


# Figure S10. Funnel Plot of primary outcome (Mortality, 12-36 months)


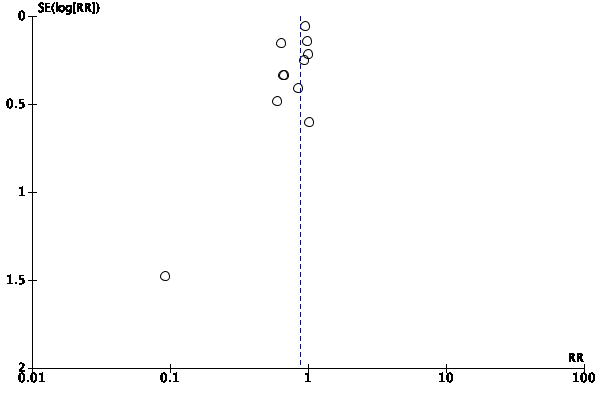

Supplement: Supplementary file 1 — Supplementary Material 1 [file 12877_2023_4036_MOESM1_ESM.docx]
